# The plant virus transmissions database

**DOI:** 10.1099/jgv.0.001957

**Published:** 2024-03-05

**Authors:** Dick Peters†, Emilyn E. Matsumura, Paulien van Vredendaal, René A.A. van der Vlugt

**Affiliations:** 1 Laboratory of Virology, Wageningen University and Research, Wageningen, Netherlands; 2 Wageningen Library, Wageningen University and Research, Wageningen, Netherlands

**Keywords:** plant viruses, plant virus transmission, plant virus database

## Abstract

Plant viruses are transmitted mechanically or by vegetative propagation, and by vectors such as arthropods, fungi, nematodes, or parasitic plants. Sources to access available information regarding plant virus transmissions are scattered and require extensive literature searches. Here, a recently created plant virus transmission database is described. This was developed to provide access to the modes of transmission and vectors of over 1600 plant viruses. The database was compiled using over 3500 publication records spanning the last 100 years. The information is publicly accessible via https://library.wur.nl/WebQuery/virus and fully searchable by virus name, taxonomic position, mode of transmission or vector.

**Impact Statement**

This paper adds and brings together important information on the transmission modes and reported vectors of plant viruses, a group of plant pathogens responsible for significant crop losses worldwide. This publicly available database allows a wide audience, also from outside of the scientific community, easy access to scientific information that spans a period of over 100 years of research within the plant virology field.

## Introduction

Over the last 120 years, a very large number of plant viruses have been reported and described, and many of those can have a significant impact on plant and crop quality and yield. Since their first descriptions, a prime focus of plant virus research has been on the way plant viruses are transmitted. Elucidating plant virus transmission mechanisms and routes, and identifying virus vectors is crucial for understanding their epidemiology and ecology, and for implementing adequate virus control measures. Over the last 100 years, countless scientific papers have been published describing ‘new’ viruses, their host plants, their associated symptoms and means of transmission. However, much of that biological information dates back to well over 40 years ago and, despite the fact that we now live in a digital age, many of these older papers may not be known or accessible to the plant virology community around the world. Bibliographic databases like Scopus, Web of Science or Google Scholar can assist in finding specific information on particular viruses but these often require combining multiple search sessions. Additionally, available (plant) viral databases like ViralZone (viralzone.expasy.org), DPV Web (www.dpvweb.net) or NCBI Virus (https://www.ncbi.nlm.nih.gov/labs/virus/vssi/#/) hold substantial amounts of data on virus genomes and taxonomy, but information on the modes of virus transmission and vectors has not been compiled anywhere or is scare. Thus, accessing this information for some viruses, through the available databases, can be difficult and time consuming.

## Methods

The plant virus transmissions database has been developed as a project aiming to provide a wide audience of users with comprehensive information on the modes of transmission and vectors of described plant viruses. Over the last ten years, more than 3500 publications covering over 100 years of plant virus research were screened for relevant information related to the modes of transmission and vectors of around 1600 plant viruses.

Per individual virus, publications describing its mode(s) of transmission or vector(s), preferably experimentally verified, were collected by searching available databases as well as the physical library collection at Wageningen University library.

The retrieved information was initially compiled in a central Excel datasheet and coupled to the respective bibliographic source(s). Additionally, taxonomic information on the virus family and genus for each virus, as published in the corresponding publication(s), was included in the Excel datasheet.

After enriching the reference information using metadata from Crossref and OpenAlex, all virus metadata from the Excel file and literature references from a Word file were converted and stored in an Oracle database. The data from Oracle was then indexed in SOLR to provide an easy search and filter interface to end-users. A simple edit interface was built to be able to add and edit viruses/references data stored in the Oracle database.

## Results

Screening over 3500 publications resulted in information of over 1600 plant virus species on their modes of transmission and vectors. The oldest bibliographic references date back to the 1920s, describing the transmission of a sugarcane virus [1] or cowpea mosaic virus by the bean leaf beetle [2]. The newest publications were included from early 2023.

Each virus’s record lists the virus name and virus family and genus status as published in the referred papers. Modes of transmissions for each virus as reported in the various publications were scored in different categories: seed-borne/not seed-borne, sap transmissible/not sap transmissible, soil-borne, water-borne and vegetatively transmissible (see Table 1). Modes of transmission were only scored for a virus when explicitly reported and these may overlap, meaning that a particular virus can e.g. be seed-borne and sap transmissible. For a very substantial number of virus species no (experimental) information was reported on the mode(s) of transmission. Among the viruses included in this database, nearly half of them (794) were reported to be transmitted by vegetative propagation and 636 were reported to be sap transmissible. Much smaller numbers of viruses were reported to be seed-borne (193) or not seed-borne (141) and only 31 were reported to be water-borne and 67 were soil-borne. The differences in virus numbers across the included categories may simply reflect the differences in focus between the studies reporting these modes of transmission. When original host plant species were reported, these were checked in The Standard Cyclopedia of Horticulture [3] and vegetative propagation was noted if applicable.

**Table 1.** Numbers of plant viruses reported for each transmission category included in the database

Mode of transmission	no. of viruses
Seed-borne	193
Not seed-borne	141
Sap transmissible	636
Not sap transmissible	114
Soil-borne	67
Water-borne	31
Vegetatively propagated	794

Next to the different transmission categories listed in Table 1, viruses were also scored for the type of vector(s) reported but these two ways of categorization have no direct relation to each other. Table 2 lists the number of viruses and virus genera for which a distinct vector was reported in literature. For only slightly more than half of the viruses (827) currently included in the database, vector transmission was reported. Aphids were reported as vector for the majority of them (328 virus species), followed by whiteflies (180 virus species) and leafhoppers (66 virus species). Several virus genera have more than one reported vector (e.g. the genus *Potexvirus* has aphids, whiteflies, bumblebees, grasshoppers, mites and even a synchytrid fungus as reported vectors), while 36 virus genera have no reported vector(s). It should be noted that for nearly all virus genera, for which a vector or vectors were reported, a significant number of virus species in these genera lack any information on possible vectors. In other words, the reported vectors for each virus genus do not apply for all virus species within the same genus.

**Table 2.** Vectors reported for the plant virus genera included in the Plant virus transmissions database

Vector reported	# of viruses	# of genera	Virus genera
Aphid	328	38	*Alfamovirus, Anulavirus, Babuvirus, Badnavirus, Betacarmovirus, Betanucleorhabdoviru, Bromovirus, Capillovirus, Carlavirus, Caulimovirus, Cheravirus, Closterovirus, Comovirus, Cucumovirus, Cytorhabdoviru, Enamovirus, Fabavirus, Ilarvirus, Luteovirus, Macanavirus, Macluravirus, Mandarivirus, Menthavirus, Nanovirus, Panicovirus, Polerovirus, Potexvirus, Potyvirus, Sadwavirus, Sequivirus, Sobemovirus, Tobamovirus, Torradovirus, Tritimovirus, Tymovirus, Umbravirus, Vitivirus, Waikavirus*
Whitefly	180	14	*Alphacarmovirus, Begomovirus, Capulavirus, Carlavirus, Citlodavirus, Crinivirus, Ilarvirus, Ipomovirus, Nepovirus, Ourmiavirus, Potexvirus, Solendovirus, Torradovirus, Tymovirus*
Leafhopper	66	17	*Alphanucleorhabdovirus, Becurtovirus, Cytorhabdovirus, Gammanucleorhabdovirus, Marafivirus, Mastrevirus, Olivavirus, Phytoreovirus, Potyvirus, Sobemovirus, Soymovirus, Tenuivirus, Topilevirus, Topocuvirus, Tungrovirus, Turncurtovirus, Waikavirus*
Mite	51	15	*Allexivirus, Cilevirus, Dichorhavirus, Emaravirus, Higrevirus, Idaeovirus, Nepovirus, Ourmiavirus, Poacevirus, Potexvirus, Potyvirus, Rymovirus, Sobemovirus, Trichovirus, Tritimovirus*
Beetle	36	8	*Betacarmovirus, Bromovirus, Carmovirus, Comovirus, Machlomovirus, Nepovirus, Sobemovirus, Tymovirus*
Thrips	31	6	*Alphacarmovirus, Ilarvirus, Machlomovirus, Nepovirus, Orthothospovirus, Sobemovirus*
Mealybug	30	6	*Ampelovirus, Badnavirus, Gammacarmovirus, Olivavirus, Velarivirus, Vitivirus*
Plasmodiophorida	25	6	*Aureusvirus, Benyvirus, Bymovirus, Furovirus, Pecluvirus, Pomovirus*
Planthopper	24	7	*Alphanucleorhabdovirus, Anulavirus, Cytorhabdovirus, Fijivirus, Grablovirus, Oryzavirus, Tenuivirus*
Nematode	23	7	*Cheravirus, Dianthovirus, Ilarvirus, Nepovirus, Sequivirus, Stralarivirus, Tobravirus*
Olpidiaceae	21	8	*Alphanecrovirus, Betanecrovirus, Dianthovirus, Galantivirus, Gammacarmovirus, Ophiovirus, Tombusvirus, Varicosavirus*
Bumblebee	3	2	*Potexvirus, Tobamovirus*
Grasshopper	2	2	*Nepovirus, Potexvirus*
Mirid	2	1	*Sobemovirus*
Leafhopper	1	1	Unassigned in *Rhabdoviridae*
Fungus	1	1	*Cucumovirus*
Synchytriaceae	1	1	*Potexvirus*
Weevil	1	1	*Bromovirus*
Cuscuta	1	1	*Tobamovirus*
No vector reported		36	*Alphachrysovirus, Alphaendornavirus, Alphapartitivirus, Amalgavirus, Avenavirus, Banmivirus, Betapartitivirus, Bevemovirus, Blunervirus, Bluvavirus, Brambyvirus, Cavemovirus, Citrivirus, Divavirus, Eragrovirus, Foveavirus, Goravirus, Hordeivirus, Lolavirus, Maculavirus, Maldovirus, Pelarspovirus, Petuvirus, Platypuvirus, Polemovirus, Prunevirus, Pteridovirus, Ravavirus, Robigovirus, Rosadnavirus, Roymovirus, Sustrivirus, Tepovirus, Vaccinivirus, Wamavirus, Zeavirus*

The database is searchable either through directly entering a name of a particular plant virus, plant virus genus or family or virus vector in the general search box at the top of the page or through the filters on the left of the webpage. These filters operate either at the level of virus genus, the vector organism or the modes of transmission. When selecting any of these filters, an alphabetical list of plant viruses that comply with the respective filter is displayed. The active filter is visible in the green box at the top of list. The total number of viruses in the active list is shown below the green box as ‘Records’. The list can be subsequently sorted by name, genus or family through the blue box on the top right of the list.

Upon activating the first filter, additional (subsequent) filters become available in the different categories on the left allowing further refinement of the search. Each of the applied filters is indicated by an additional green box. Filters can be individually closed by clicking on the ‘x’ in the corresponding green box.

At the top of the active list, the different modes of transmission are visible and a ticked box quickly indicates which of these modes has been reported for each listed virus.

When clicking on the downward arrow on the left of a particular virus, more detailed information is displayed on its virus taxonomic position and the main reported vectors and modes of transmission. The taxonomic position was brought in line as much as possible with the current ICTV classifications. For clarity however, the virus names were maintained in the database as originally reported, and none of the recently assigned binomial virus names were used, but this can be implemented in future updates.

**Fig. 1.**
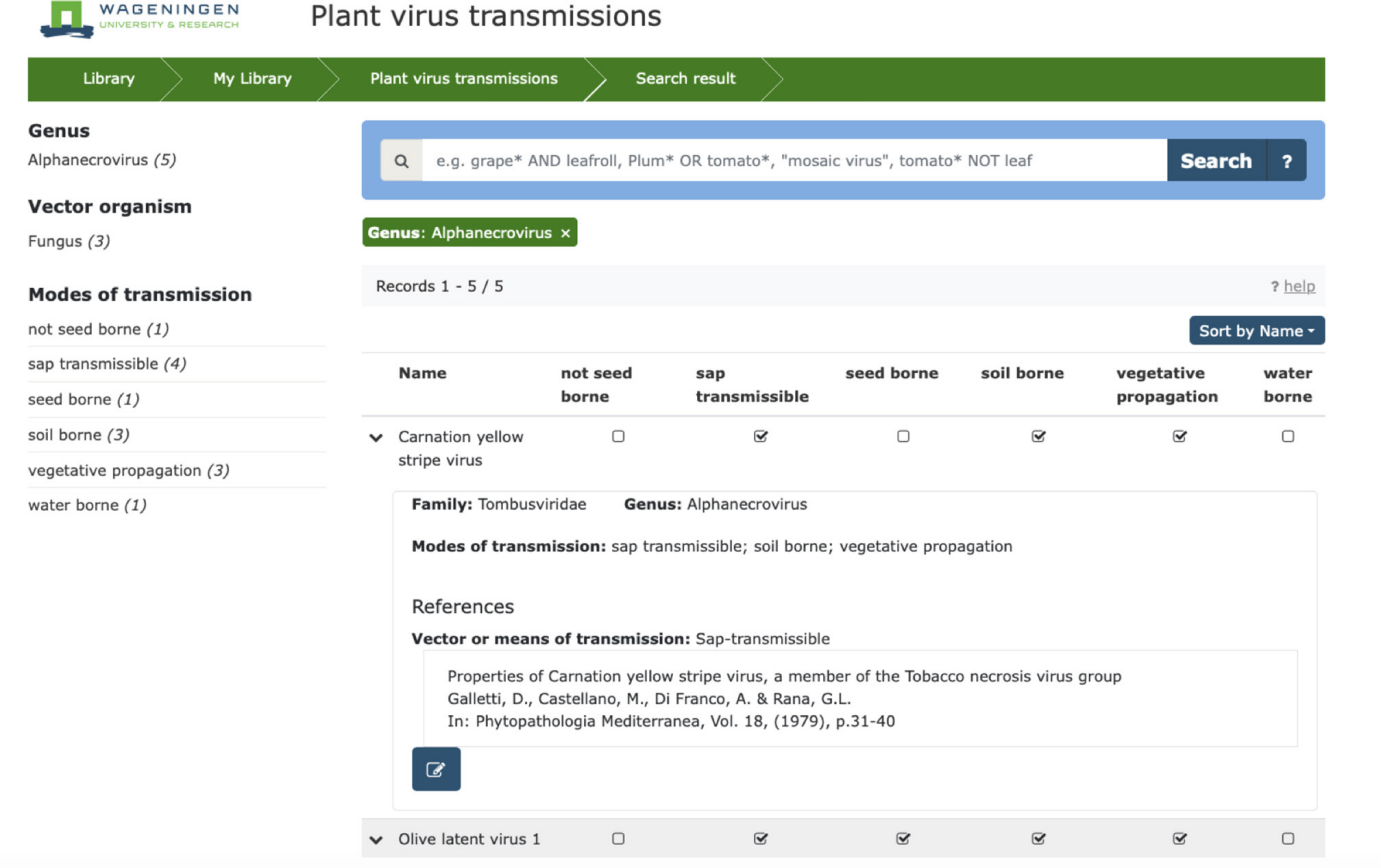
Screen shot from the plant virus transmissions database website showing an example of output. When users select a specific filter (in this case, genus *Alphanecrovirus*) and showing an example of information displayed when a specific virus is selected from the filter list (in this case, carnation yellow stripe virus). Displayed information shows the virus family, genus and reported modes of transmission, as well as the reference list from where the information were retrieved.

Each virus record also lists the bibliographic information for the particular publications from which the information was compiled, and for each publication it briefly summarizes the reported vector(s) and means of transmission (Fig. 1).

A bold and underlined reference title indicates that a direct link to an electronically accessible reference is available and when clicking on it, users are redirected to the original publication at the publisher’s website. Direct access will however depend on the possible open access status of the publication or the permissions of the user, i.e. through institutional libraries.

## Discussion

The plant virus transmissions database, currently containing over 1600 plant viruses, was constructed to provide free, web-based access to reported information of the modes of transmissions and vectors of these viruses. Although quite substantial, the database also reveals that many gaps in information on the means of transmission or possible vectors of these plant viruses still exist. For agriculturally important plant viruses, their modes of transmission and possible vectors have often been well studied but for more than half of the viruses listed here, this information remains unreported.

For 36 virus genera, no vector has been reported, while for nearly all other virus genera the means of transmission or vector(s) have been experimentally proven for only a limited number of virus species within each genus. Given the growing problems with plant virus outbreaks worldwide, a better understanding of their epidemiology, including their means of transmission and possible vectors, is needed for adequate and timely control of these outbreaks. The database also shows that nearly half (794) of the viruses reported here are transmitted through vegetative propagation of plant and planting material. Given the current worldwide trade of plants and planting material, this emphasizes the need for adequate and reliable indexing and diagnostic methods to control the unnoticed and unwanted spread of these viruses.

Although not quantified, many of the older references often focus on the biological info, including host range, modes of transmission and possible vectors, while more recent literature focuses on molecular data. In the current ‘Ecogenomics’ age, many new plant viruses are described solely based on the discovery of genomic data, often through High Throughput Sequencing technologies and biological information on their means of transmission or possible vectors, or even infectious nature, is missing. Nevertheless, this type of information is crucial in developing adequate control measures for these viruses.

But not only these newly discovered viruses may be of agricultural and economic importance, but also ‘old’ viruses may become a problem again. For this reason, the information in this database is equally important for parties involved in phytosanitary regulations like European Food Safety Authority (EFSA), European Plant Protection Organisation (EPPO) and National Plant Protection Organisations (NPPOs). And with the growing applications of genetically modified organisms (GMOs), here to be specified as genetically modified plant viruses for e.g. agricultural of medical applications [4,5], the importance of the database extends to bodies involved in GMO, biosafety and biosecurity regulations.

It should be noted that the information in this database was taken ‘as is’ from the retrieved publications and reflect the taxonomic classification at that time. The taxonomic classification listed for each virus may therefore not reflect the latest taxonomic classifications in the Master Species List (MSL) as published by the International Committee on the Taxonomy of Viruses (ICTV: https://ictv.global/msl). Viruses in the database may not be included in the MSL or may be included in the current taxonomy under a different name.

In its present form it provides a more centralized access to this information for the plant virus community and others interested. We will strive to regularly update the plant virus transmissions database with new information according to new publications on plant virus transmission and in this respect we welcome contributions from the plant virus community and other users. We realize that the information may not be complete. We welcome additional information as well as corrections for any apparent mistakes. Please contact us at plantvirustransmissions@wur.nl.

## Abbreviations

DPV: Descriptions of Plant Viruses
ICTV: International Committee on the Taxonomy of Viruses
NCBI: National Centre for Biotechnology Information
